# Ecosystem Microbiome Science

**DOI:** 10.1002/mlf2.12054

**Published:** 2023-01-11

**Authors:** Yong‐Guan Zhu, Dong Zhu, Matthias C. Rillig, Yunfeng Yang, Haiyan Chu, Qing‐Lin Chen, Josep Penuelas, Hui‐Ling Cui, Michael Gillings

**Affiliations:** ^1^ Key Laboratory of Urban Environment and Health, Institute of Urban Environment Chinese Academy of Sciences Xiamen China; ^2^ State Key Laboratory of Urban and Regional Ecology, Research Centre for Eco‐environmental Sciences Chinese Academy of Sciences Beijing China; ^3^ Institute of Biology Freie Universität Berlin Berlin Germany; ^4^ Berlin‐Brandenburg Institute of Advanced Biodiversity Research (BBIB) Berlin Germany; ^5^ State Key Joint Laboratory of Environment Simulation and Pollution Control, School of Environment Tsinghua University Beijing China; ^6^ State Key Laboratory of Soil and Sustainable Agriculture, Institute of Soil Science Chinese Academy of Sciences Nanjing China; ^7^ Faculty of Veterinary and Agricultural Sciences The University of Melbourne Melbourne Victoria Australia; ^8^ CSIC, Global Ecology Unit CREAF‐CSIC‐UAB Bellaterra Catalonia Spain; ^9^ CREAF Cerdanyola del Vallès Catalonia Spain; ^10^ ARC Centre of Excellence for Synthetic Biology, and Department of Biological Sciences Macquarie University Sydney New South Wales Australia

**Keywords:** dynamics, ecosystem, global change, interactions, microbiome

## Abstract

The microbiome contributes to multiple ecosystem functions and services through its interactions with a complex environment and other organisms. To date, however, most microbiome studies have been carried out on individual hosts or particular environmental compartments. This greatly limits a comprehensive understanding of the processes and functions performed by the microbiome and its dynamics at an ecosystem level. We propose that the theory and tools of ecosystem ecology be used to investigate the connectivity of microorganisms and their interactions with the biotic and abiotic environment within entire ecosystems and to examine their contributions to ecosystem services. Impacts of natural and anthropogenic stressors on ecosystems will likely cause cascading effects on the microbiome and lead to unpredictable outcomes, such as outbreaks of emerging infectious diseases or changes in mutualistic interactions. Despite enormous advances in microbial ecology, we are yet to study microbiomes of ecosystems as a whole. Doing so would establish a new framework for microbiome study: Ecosystem Microbiome Science. The advent and application of molecular and genomic technologies, together with data science and modeling, will accelerate progress in this field.

## INTRODUCTION

**Figure 1:**
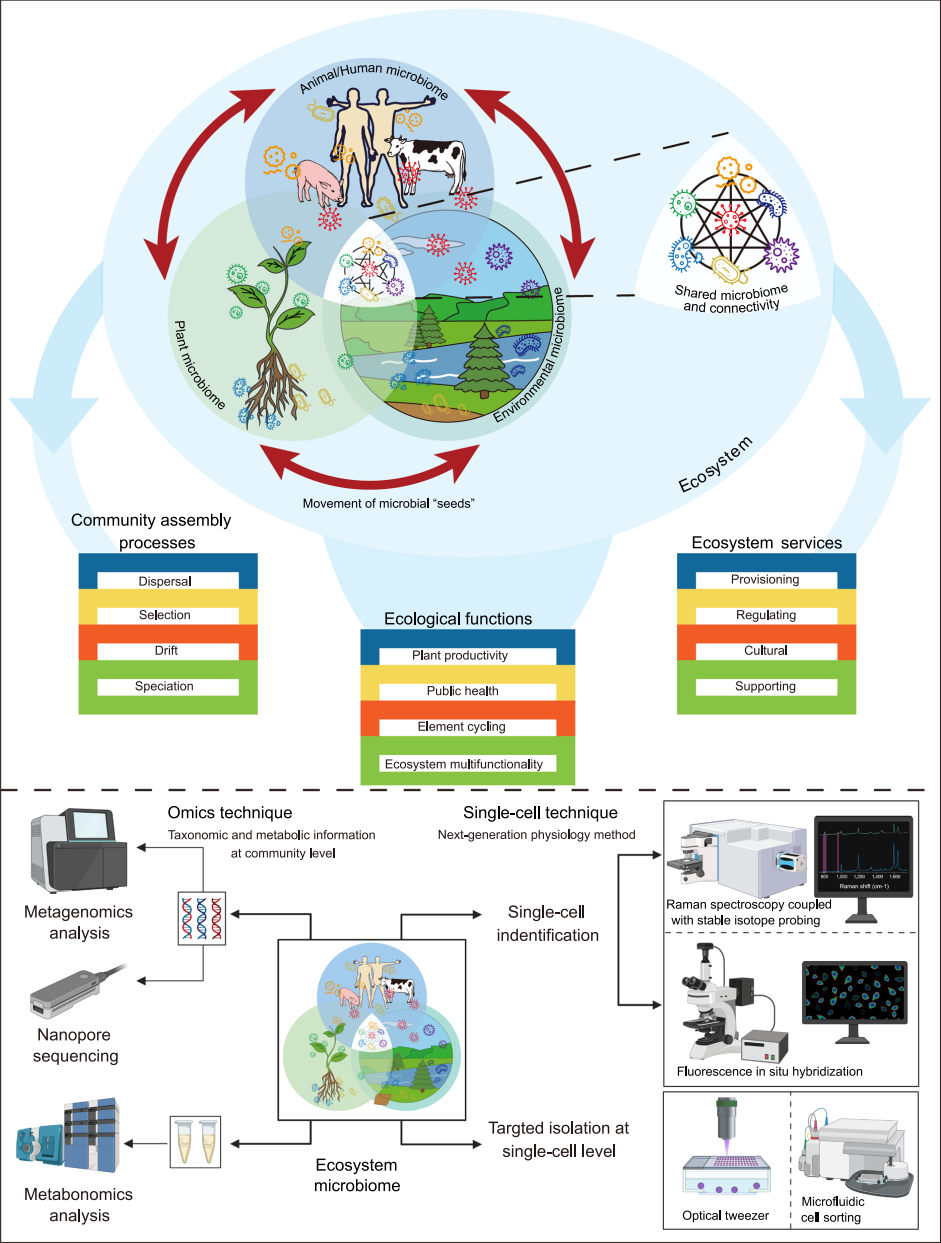
The concept of ecosystem microbiomes and a technique map to briefly show how to study microbial genomes and functions in whole ecosystems. In general, omics methods could provide taxonomic and metabolic information at the community level. The recently developed functional Raman spectroscopy‐based single‐cell technology as a next‐generation physiology method could provide information on microbiome function at the single‐cell level.

Human prosperity relies on ecosystem functioning including production of food, fiber, and fuel. Ecosystem ecology primarily addresses the exchange of energy and materials between organisms within an ecosystem and with the abiotic environment within which an ecosystem is functioning. It studies the interactions between organisms and their physical environment as an integrated system^1^. Such studies have dealt primarily with macro‐organisms and predominantly focused on vascular plants. With the advent of modern genomic and molecular techniques, such as the second‐ and third‐generation sequencing technologies as well as the metabolomics, many fascinating discoveries were made on microbial diversity and their interactions. Microbial community structures and basic patterns of the composition and dynamics of microbial communities have now become feasible even at global scales^2^, ^3^. In addition to the omics techniques, next‐generation physiology approaches such as Raman microspectroscopy represents a potentially game‐changing technology for microbial ecology^4^, ^5^. The Raman spectrum of a cell is a unique fingerprint of its chemical composition and contains information on its taxonomic identity and physiological state^5^. More importantly, this technique is nondestructive and allows downstream analyses such as sorting, sequencing, or cultivation of taxa of interest^6^, ^7^. All these techniques have rapidly expanded, increasing the realization that microorganisms are playing a central part of all ecosystem ecological functions and services (Figure 1). On the one hand, microbes influence plant performance and primary production, mediating tolerance to biotic stresses and fostering nutrient acquisition, and on the other hand, they play central roles in virtually all biogeochemical processes^8^, ^9^. These processes occur simultaneously and are often colocated, and yet, are typically studied in isolation. For example, there are many studies on the effects of the soil microbiome on plant performance, but it is unclear how other microbial processes at the ecosystem level affect feedbacks on plant growth and vice versa. Since microbial activities are interconnected, biological mechanisms operating at one level of biological organization can affect functioning at higher organizational levels^10^. As a result, ecosystem processes across entire microbiomes should be examined simultaneously across organizational levels to uncover intricate linkages and feedbacks. This is the essence of what we are calling Ecosystem Microbiome Science—the simultaneous examination of microbial genomes, functions, and their interactions in whole ecosystems.

The term microbiome refers to all microbes, including archaea, bacteria, fungi, protists, and viruses, that are associated with an animal or plant host or occupy an environmental compartment such as soil and water. With the advent of molecular and genomic tools and data science, it is now much easier to capture and understand the complexity of microbial systems. Microbiome studies often examine the composition and function of the microbiome in individual hosts or in a sample taken from a particular environment. However, due to the lack of systematic thinking and accurate microbial tracing technology, microbiome studies are rarely integrated with measurements of the pools and fluxes of matter and energy in ecosystems. Similarly, the spatial and temporal dynamics of microbiomes is rarely examined. Therefore, we define the ecosystem microbiome as the consortia of microbes that reside within a given ecosystem (e.g., tropical forest, human body, or paddy field). Ecosystem Microbiome Science addresses the interactions between microbiomes and the environment and the movement and functions of microbiomes within an integrated ecosystem. Based on previous results and current knowledge, four general questions for Ecosystem Microbiome Science studies will be discussed in the paper: (1) distribution and movement of microbiomes within an integrated ecosystem; (2) connectivity of ecosystem microbiomes; (3) temporal dynamics of ecosystem microbiomes; and (4) applications of Ecosystem Microbiome Science.

## DISTRIBUTION AND MOVEMENT OF MICROBIOMES WITHIN AN INTEGRATED ECOSYSTEM

Traditionally, characterization of an ecosystem is achieved by the analysis of the fluxes of energy and materials through organisms and the physical environment and by cataloguing biodiversity (mostly of macroorganisms) within an ecosystem. However, we believe that such a characterization is becoming increasingly insufficient for understanding the properties and functions of an ecosystem. Although there are numerous studies on the soil microbiome and emerging studies on the plant microbiome, we still know little about these microbiomes at the ecosystem level. For example, do different ecosystems have signature microbiomes/microbial taxa/keystone species? How do ecosystems differ in terms of microbial connectivity among different components? And does it matter? What are the driving forces of microbiome exchange between different components of an ecosystem? To answer all these questions, we should analyze the spatiotemporal dynamics of microbiomes at an ecosystem level, and we need to study the connectivity of microbiomes between different ecosystem components.

Assessing the distribution of microbiomes within an ecosystem requires comprehensive sampling and sequencing, both spatially and temporally^11^. By taking samples from highly accessed urban surfaces and parks in New York City, Afshinnekoo et al.^12^ found that 1688 bacterial, viral, archaeal, and eukaryotic taxa inhabited the area. The microbiota were enriched for genera associated with skin, showing transfer of human microbiota into the environment. They also showed that bacterial signatures can match the history of an extreme climate event, such as the appearance of marine‐associated bacteria in a hurricane‐flooded station. Using bioinformatics and machine learning, microbial genomics data can generate fingerprints of cities based on urban microbiome compositions^13^. Such studies and data sets may shed light on the spread of human‐derived microbes and help develop surveillance tools for human infectious diseases worldwide.

The “terroir” concept used in wine‐making^14^ is another example of the role of microbiomes within an ecosystem^15^. In a comprehensive study of the soil and vine microbiome, Zarraonaindia et al.^16^ found that while vine‐associated microbiomes were affected by an array of diverse factors, the majority of organ‐associated microbial taxa originated from soil and their distribution reflected both the impact of local biogeographic factors and how the vineyard was managed. This suggests that soil serves as a major source of vine‐associated bacteria and influences the grape‐associated microbiome important for wine‐making. We can further infer that signature ecosystem microbiomes (i.e., soil‐ and vine‐associated microbiomes) and biogeographic factors affecting them may contribute to a regional terroir of the wine. Beyond this “terroir” example, the plant and soil microbiome will impact postharvest processes for many crops. The influence of the ecosystem microbiome likely extends far into the agri‐food chain; for example, soil microbiome elements may determine food storage traits or influence food nutritional value^14^.

In ecosystems, microbes can move as entire communities^17^, not just as individual species or phylotypes, the latter movement being well captured in current meta‐community theory^18^. Since parts of the environment are colonized by microbes, and these parts can be moved by a range of processes (including gravity and water flows), the associated microbial communities are moved as well. The encounter and mixing of previously separated microbial communities is community coalescence^18^, ^19^. This concept includes the merging of entire communities and their environments (e.g., in aquatic environments, the mixing of saline water and freshwater). An immediately obvious example is litterfall, whereby the leaf microbiome encounters the soil microbiome on a massive scale. Theory and empirical evidence also suggests that communities, upon coalescence, can maintain a certain degree of coherence, highlighting the importance of studying such microbiome fluxes at the ecosystem scale, incorporating the microbiome endmembers (i.e., pools), fluxes, and resulting community coalescence phenomena^20^.

## CONNECTIVITY OF ECOSYSTEM MICROBIOME

### Trophic linkages of microbiomes within an integrated ecosystem

**Figure 2:**
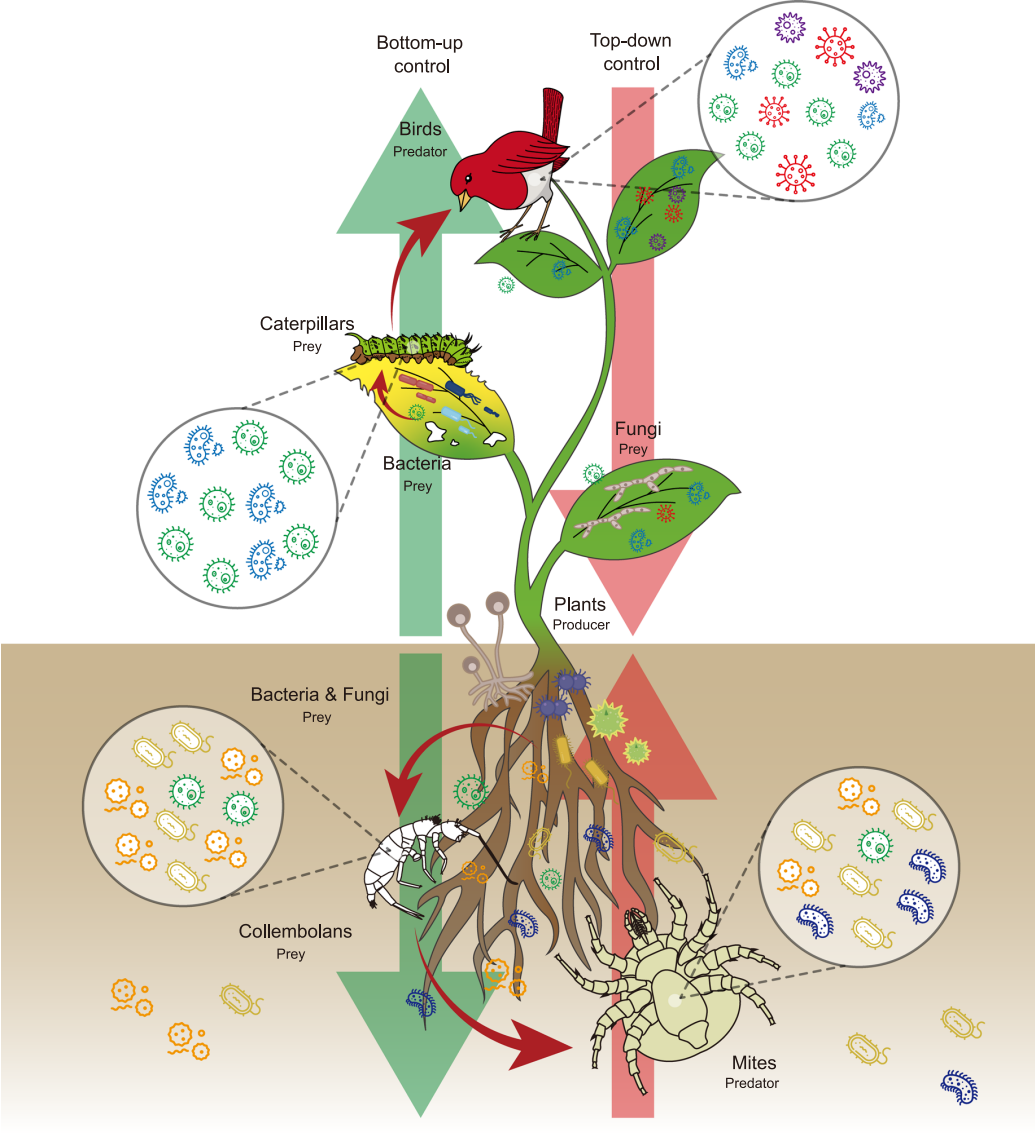
The connectivity of the ecosystem microbiome. Trophic regulation including bottom‐up and top‐down control of the ecosystem microbiome is the key ecological process that links to the flow of materials and energy in the ecosystem.

Food webs are depictions of feeding relationships with an ecosystem (Figure 2). Trophic relationships regulate key ecological processes such as the flow of materials and energy^21^, ^22^. The most frequently studied components of food webs, animals and plants, are always colonized by microorganisms that play important roles in the health and biogeochemistry of their hosts^23^, ^24^. Consequently, while these microorganisms are tightly connected to the food web in an integrated ecosystem^25^, they are largely ignored, except for their roles as degraders.

Different organisms have distinct microbiomes, suggesting that these microorganisms play different roles in ecosystems. The specificity of these interactions means that extinction of hosts could potentially lead to a decline in the overall microbial diversity and associated functions, even before these microorganisms are discovered. It has been suggested that the transfer of microorganisms in the food web plays an important role in the health of animals and plants in an integrated ecosystem^26^. The microbiome of organisms at higher trophic levels can be a subset of those at lower trophic levels in the food web and in the environment^27^. This suggests that organisms higher up the food chain can acquire their microbiome from organisms lower down. However, it is not fully understood which microbes can be transferred between organisms at different trophic levels. Broadly, the trophic dynamics of the microbiome is controlled by two biotic factors within ecosystems: diet‐driven (bottom‐up) and consumer regulation (top‐down). Effects of food and predators on the structure and function of food web have been widely studied, such as predator control of nutrient cycling^28^. As expected, they have direct/indirect effects on the structure and function of microbiomes in the food web.

### Bottom‐up and top‐down control of the ecosystem microbiome

Diet has a vital influence on the composition and structure of an individual animal microbiome^29^, ^30^. For example, an antibiotic‐treated diets could not only alter the gut microbiome of cattle but could also affect the microbiome of nontarget animals such as dung beetles and affect the ecosystem services that they provide^31^. In addition, prey could alter the microbial community structure of a predator^32^. In a zebrafish experiment, the microbiome could be dispersed between species^33^, suggesting that a change of community structure in the prey might affect the dynamics of the microbiome in the whole ecosystem. Moreover, food could act as a vector for microbiome transfer in the food web. In a soil–plant–caterpillar study, soil microbiomes could be transmitted into insect microbiomes via ingested plants. Movement of insects could carry acquired microorganisms into other organisms’ microbiomes^34^. By understanding microbiome dynamics at the ecosystem level, we could better reveal the effects of diet on ecosystem processes and functions.

Organisms at higher trophic levels directly alter the microbiome of prey via predation. There are two scenarios regarding the impact of predation on the microbiomes of prey. First, if the prey is ingested or dead, the niche associated with prey will shift or predation will produce an unrecognized route of bacterial transmission. In a snail‐grazed mesocosm study, grazing by snails increased fungal community heterogeneity in the phyllosphere and could lead to ecosystem fragmentation^35^. A recent field study also indicated that insect herbivory could alter endophytic bacterial diversity patterns within a native plant host^36^. Second, if the prey is not ingested, its microbiome may also be altered under the pressure of predation. Predation stress could reduce species richness and alter the metabolic repertoire of bacteria in the microbiome of prey^37^. This is likely because prey can alter their behavior, diet, and habitat to avoid being captured.

Interactions between species could alter the effects of predators on ecosystem microbiomes. For example, cascading effects could magnify the impact of top predators, affecting microbiomes of lower trophic levels in the food web by altering the behavior, morphology, and population size of organisms at other trophic levels. Effects of one predator extinction on the diversity of a microbiome in an ecosystem might be reduced due to trophic redundancy. Although related studies regarding trophic redundancy in animal microbiomes are scarce, some evidence has been emerging from community ecology. Construction of communities with four trophic levels^38^ revealed that trophic redundancy could decrease the vulnerability of ecosystems to extinction of predators due to biodiversity loss^38^.

## TEMPORAL DYNAMICS OF THE ECOSYSTEM MICROBIOME

The dynamic interplay between organisms and resource supply determines succession in ecosystems, these being directional changes in structure and function. Succession has been understood for many years as an ordered sequence of communities building to a climax community or as a predicted community composition dictated by the abiotic and biotic environmental conditions within the biome^39^. The structure and functioning of ecosystem microbiomes also change over time in primary successions (in newly created habitats) and in secondary successions (with survival of residuals, e.g., after a disturbance such as fire, drought, typhoon, or landslide). For example, de Araujo et al.^40^ investigated the soil microbiome along a gradient of Cerrado savannah, including grass, grass and shrub, shrub and tree, and tree‐dominated zones. They found that complexity of the microbiome increased towards the tree‐dominated climax and that the highly interconnected microbiome played a vital role in maintaining ecosystem performance. Moreover, microbial assembly could be shifted during the succession, for example, the soil microbial community composition was initially governed by stochasticity, but there was a progressive increase in deterministic selection as succession proceeded in a salt mash chronosequence spanning 105 years^41^. Despite these efforts, our understanding of succession of microbiomes during ecosystem development is still in its infancy, especially at an ecosystem level, and it is not clear if the climax communities are the same for the microbiomes in similar compartments of different ecosystems. Future studies will be needed to go beyond the soil microbiome, to include the microbiome in other ecosystem compartments. Plant roots, leaf surfaces, and soil invertebrates are all likely to have unique climax communities that interact with soil, water, and atmospheric microbiomes. Understanding connectivity, cosuccession, and coevolution of microbiomes across ecosystems will help us manage their influence on ecosystem functioning.

Microbiomes can exist in alternative stable states, similar to alternative vegetation landscapes, such as savannah versus forests^42^. These alternative states can depend on the order of species arrival to the new environment (priority effects) or on the selective removal of keystone species. Once established, communities have positive feedbacks that can build resistance and resilience to change in response to further disturbance. The changes in microbiome communities depend on predisturbance composition, source pools for recolonization postdisturbance, and type of disturbance, but they always occur due to the disappearance of some species and the appearance of others. Disappearances may arise from local extinction or emigration out of the area, and appearances may arise from speciation or immigration into the area. The best model for these phenomena is the human microbiome and its effect on health^43^, but similar effects must be happening in all other organisms whose microbiota are affected by human activity.

Species turnover can be high among microbes, particularly in bacteria, which can speciate rapidly due to shorter generation time and horizontal gene transfer (HGT)^17^, ^44^. It has been suggested that HGT could accelerate bacterial adaptation by vectoring ecologically important traits or reducing selective constraints on the spread of genetic variation^45^, ^46^. Therefore, HGT is an evolutionary force that facilitates the spread of nonselected genetic variation and expands the adaptive ability of microbial populations, which will substantially impact the ecosystem microbiome and lead to new uncertainties in terms of the use of traditional ecological theories and models to explore microbial succession and community assembly at the ecosystem level^47^, ^48^.

## APPLICATIONS OF ECOSYSTEM MICROBIOME SCIENCE

### Links between the ecosystem microbiome and emerging infectious diseases (EIDs)

Individuals disperse more than 300 million bacterial cells into the environment on a daily basis, with human skin being the dominant contributor to the microbiome of built environments^49^. However, industrialization and urbanization have resulted in a substantially less diverse human microbiome^50^, while anthropogenically induced changes, such as global warming and eutrophication, have also reduced environmental microbial diversity^51^, ^52^. As a consequence, many beneficial microbes needed for health are disappearing^53^. Perturbations to the human microbiome are increasingly being associated with allergies, cancer, type II diabetes, depression, vascular, inflammatory, and neurological and cognitive disease. Such conditions can accelerate the likelihood and impact of global pandemics.

EIDs are threatening global health and stability. Many EIDs are deeply rooted in the environment, and changes in ecosystem microbiomes play a crucial role in their emergence. The cascading effects of human‐induced changes on EIDs have been widely observed, such as land conversion and spillover of pathogens through biodiversity loss and changes in human–nature contact^54^, ^55^. This is particularly true with zoonoses originating from wildlife, such as COVID‐19. For example, the risk of zoonotic EIDs is elevated in tropical regions with high biodiversity, but experiencing land‐use changes^56^.

Cascading effects on ecosystem microbiomes are also caused by chemical pollution. Various chemicals are known to alter microbiomes and their metabolic capabilities^57^. The herbicide glyphosate can eliminate beneficial bacteria such as *Bifidobacterium adolescentis* and *Bacillus badius*, while stimulating pathogenic bacteria such as *Salmonella enteritidis* and *Clostridium perfringens* ^58^. With rapid global warming, permafrost thaw and glacial retreat in polar and alpine regions could release pathogens and viruses that have been trapped and preserved for tens to hundreds of thousands of years. *Pithovirus sibericum*, *Mollivirus sibericum*, and Pandoraviruses are some of the 30,000‐year‐old icosahedral DNA viruses isolated from Siberian permafrost. These are capable of infecting unicellular protists such as Acanthamoeba, suggesting that global warming or industrial exploitation of circumpolar regions might lead to unexpected threats to human or animal health^59^, ^60^. Noroviruses and coronaviruses often adsorb readily to soils and remain viable from days to years^61^, ^62^, ^63^, which poses a previously overlooked health risk. In contrast, pathogenic organisms such as *Salmonella enterica* ssp. and enterica serovar *Typhimurium‐lux* can leach through soils, suggesting that groundwater contamination from vertical movement of pathogens is also a potential risk^61^.

A comprehensive understanding of the human health risk associated with environmental microbiomes will require a holistic view that emphasizes interactions among members within microbiomes, as well as their coevolution with their neighbors and hosts. As viruses are important reservoirs of antibiotic resistance genes in the environment^64^, the practice of using antibiotics to treat complications of viral infection should be carefully reconsidered. To wholly appreciate the complex level of interspecies interaction, it is necessary to use the full arsenal of current technologies. It is also important to carefully design longitudinal studies associated with simplified, pragmatic sampling strategies.

### Conservation of the ecosystem microbiome: Microbial seed banks

**Figure 3:**
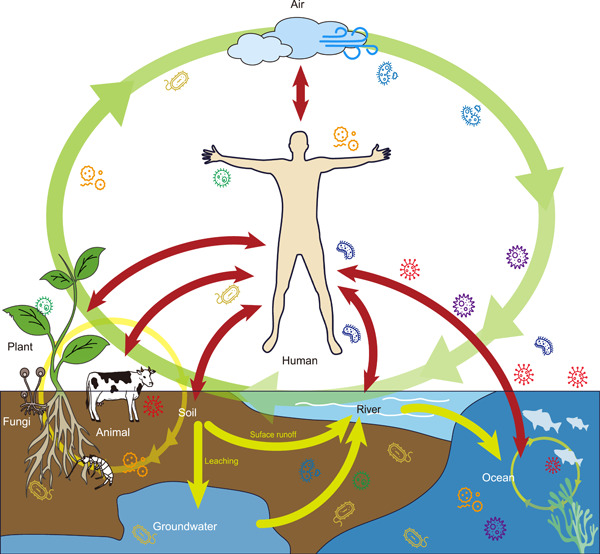
Microbial cycling between humans and ecosystems. It is generally accepted that microbes are important for human health, and novel functions of the microbiome in human health and well‐being are being increasingly recognized. Therefore, there is a need to better understand the potential connections between ecosystem and human health in an ecosystem context.

Humans are now the greatest evolutionary force on the planet^65^ (Figure 3). Our activities significantly alter ecosystems and have precipitated the 6th Mass Extinction^66^, ^67^. Microorganisms are not immune to these effects and are likely also going extinct^68^, ^69^. The best evidence for potential extinctions comes from studies of the human microbiota, where significant numbers of taxa are known to be missing from urbanized populations^43^. The loss of these microbes may promote the spread of antibiotic resistance, reduce the ability of host to resist epidemics, and may also affect host development. Therefore, the potential for microbial extinction events has led to the establishment of “seed banks” of the human microbiota, sourced from multiple cultures, but focused primarily on collections from minimally disturbed traditional populations^70^. Other microbiome banks are being established for medical, agricultural, and conservation purposes^71^, ^72^.

Most data on the diversity of microbiota have been obtained from studies on humans and a few other model species. There is, however, an increasing interest in the microbiota of other organisms, including plants, birds, and invertebrates, in addition to mammals^73^, ^74^, ^75^, ^76^, ^77^, ^78^, ^79^. This is appropriate, given that microbial extinctions are likely to affect the microbiota of all organisms directly affected by human activity. Such studies are particularly important for terrestrial plants, since most plants form symbioses with soil microorganisms^80^, ^81^. We suspect that genes in the microbiota will allow an opportunity for rapid acquisition of new phenotypes and consequently, rapid adaptation to environmental change^82^.

The microbiota are also likely to influence behavior in both humans and in animals^83^, ^84^. Conservation geneticists have been promoting the preservation of genetic diversity for some time^85^. However, if microbiota are a significant source of phenotypic and functional diversity, we risk losing this diversity with the loss of species from the microbiota. As a consequence, calls are being made for the conservation of the holobiont, that is, the macro‐organism along with its resident microbiota^84^. Such conservation must extend to both plants and animals^86^. The conservation of host species microbiota could be an essential tool for saving these hosts from extinction^87^.

## CONCLUDING REMARKS

Microbiomes from different compartments within an ecosystem are highly connected. Their roles in mediating ecosystem‐level processes and functions are determined by their physiology and by their dynamic movement and interactions with other biotic/abiotic factors in an ecosystem. We, therefore, propose that future research should embrace the entire microbiome within an integrated ecosystem to avoid fragmented views of microbiome properties, from the perspective of both community and functional traits. We posit that the tools and frameworks of ecosystem ecology can be applied and expanded to microbiome science, so that we can better understand the links between the different compartments of ecosystems. This would revolutionize our ability to understand and harness links and predict responses to anthropogenic influences, such as EIDs.
